# Bone Marrow Mononuclear Cells Administration Restore Lysophosphatidic Acid (LPA) Levels and Cellular Signaling Axis in Rats Submitted to Renal Ischemia–Reperfusion

**DOI:** 10.3390/ijms26189186

**Published:** 2025-09-20

**Authors:** Paula Mattos-Silva, Sabrina Ribeiro Gonsalez, Lucienne S. Lara, Marcelo Einicker-Lamas

**Affiliations:** 1 Laboratório de Biomembranas, Instituto de Biofísica Carlos Chagas Filho, Universidade Federal do Rio de Janeiro, Ilha do Fundão, Rio de Janeiro 21941-902, RJ, Brazil; pmattos@gmail.com; 2 Instituto de Ciências Médicas, Universidade Federal do Rio de Janeiro, Macaé 27930-560, RJ, Brazil; srgonzales@gmail.com; 3 Instituto de Ciências Biomédicas, Universidade Federal do Rio de Janeiro, Rio de Janeiro 21941-902, RJ, Brazil; lucienne.morcillo@gmail.com; 4 Centro de Pesquisa em Medicina de Precisão, Universidade Federal do Rio de Janeiro, Rio de Janeiro 21941-902, RJ, Brazil

**Keywords:** renal ischemia/reperfusion injury, bone marrow-derived mononuclear cells (BMMC), lysophosphatidic acid (LPA), LPA receptors, autotaxin (ATX)

## Abstract

Bone marrow-derived mononuclear cells (BMMCs) have shown beneficial effects on tissue repair, largely attributed to the paracrine action of bioactive mediators such as lysophosphatidic acid (LPA). This study aimed to evaluate the effects of BMMC treatment in a rat model of renal ischemia/reperfusion (I/R) injury, focusing on LPA-related molecular pathways. Male Wistar rats were divided into three groups: control; I/R, subjected to bilateral renal artery clamping for 30 min followed by 24 h of reperfusion; and I/R + BMMC, which received 1 × 10^6^ BMMCs per kidney directly into the renal capsule post-ischemia. During reperfusion, the rats were placed in metabolic cages for urine collection, renal function and protein expression. BMMC treatment did not reverse the I/R-induced increase in urine volume or decrease in glomerular filtration rate, serum potassium, or filtered sodium load. However, it prevented proteinuria, increased blood urea nitrogen, and enhanced urinary potassium excretion. Mechanistically, BMMC treatment prevented I/R-induced upregulation of LPAR1, downregulated LPAR2 and LPAR3, restored plasma LPA levels, and reduced renal autotaxin content. These results suggest that BMMCs modulate harmful LPA-related signaling and may contribute to renal protection through paracrine mechanisms in the setting of acute I/R injury.

## 1. Introduction

Acute kidney injury (AKI) is defined based on functional alterations, which are identified by the magnitude and timing of serum creatinine elevation or reduction in urine output, occurring within a period of 6 h to 7 days [1]. AKI frequently coexists with sepsis, ischemia, and nephrotoxicity, factors that complicate its diagnosis and management. Despite its clinical relevance, no effective pharmacological therapies are currently available. Notably, AKI requiring renal replacement therapy remains associated with high mortality rates, ranging from 50% to 80% over the past decade [2,3].

In this context, cell-based therapies have emerged as promising tools for kidney repair, offering a novel approach in regenerative medicine. Among the various cell types tested in preclinical models of kidney disease, bone marrow-derived mononuclear cells (BMMCs) are particularly attractive due to their accessibility, ease of handling, and potential for autologous transplantation, thereby reducing immunological complications [4,5,6,7,8,9]. We have demonstrated the paracrine interaction between bone marrow-derived stem cells and renal proximal tubule epithelial cells [10]. While earlier hypotheses emphasized differentiation and tissue engraftment, current evidence supports a predominantly paracrine mechanism of action, mediated by the release of bioactive molecules including growth factors, cytokines, peptides, microRNAs, and bioactive lipids [11,12].

Among the latter, lysophosphatidic acid (LPA)—the simplest glycerophospholipid (1-acyl-sn-glycero-3-phosphate)—has attracted attention for its pleiotropic effects in tissue injury and repair. LPA is produced by multiple enzymatic pathways, including extracellular autotaxin (ATX), phospholipase A2 (PLA_2_), and monoacylglycerol kinase [13,14]. LPA homeostasis is tightly regulated not only by its biosynthesis but also through degradation by lipid phosphate phosphatases and lysophospholipases [15]. Dysregulated LPA metabolism has been implicated in kidney injury, where elevated LPA levels contribute to tubular cell apoptosis, immune cell recruitment, and profibrotic signaling [16,17]. In vivo studies have shown that LPA levels increase in models of chronic kidney disease, such as unilateral ureteral obstruction (UUO), while exogenous LPA administration can reduce ischemia/reperfusion (I/R)-induced damage by limiting apoptosis and inflammation [18,19,20]. In vitro, LPA also exerts cytoprotective effects, reducing inflammatory signaling and promoting cell survival and proliferation [21,22,23,24,25]. These effects are mediated by specific G protein-coupled receptors (LPA1–6), which are known to be expressed in renal tissue [26,27,28].

To investigate these mechanisms in vivo, we employed the renal ischemia/reperfusion (I/R) model in rats. This model is well established for reproducing the pathophysiological features of AKI, including hemodynamic instability, tubular epithelial injury, inflammatory infiltration, and subsequent fibrotic responses [29,30]. The rat kidney offers anatomical and physiological similarities to the human kidney, such as nephron organization, glomerular filtration characteristics, and responsiveness to vasoactive mediators, thereby ensuring translational relevance [31]. Moreover, the I/R model closely mimics clinical scenarios of AKI in humans, such as those occurring during major surgery, transplantation, or septic shock [32,33,34].

Based on this background, our research question was whether BMMC therapy could modulate LPA-dependent mechanisms to promote renal recovery after I/R injury. The main objective of this study was to evaluate the effects of BMMC administration on circulating LPA levels, renal ATX expression, and LPA receptor (LPAR1–3) signaling during AKI. Specifically, we hypothesized that BMMCs would restore altered LPA homeostasis, thereby contributing to renoprotection and preservation of glomerular function in the rat I/R model.

## 2. Results

### 2.1. BMMC Treatment Restores Circulating LPA Levels and Modulates Renal LPA Signaling After I/R

General renal physiological parameters measured 24 h after renal I/R and BMMC treatment are shown in Table 1. No differences were observed in body weight, water intake, or urinary pH among the experimental groups. However, urine volume increased by approximately 50% in both the I/R and I/R + BMMC groups compared to control (CTRL: 3.7 ± 0.35; I/R: 7.70 ± 1.03; I/R + BMMC: 7.42 ± 1.06 mL/24 h; *p* < 0.05).

**Figure 1:**
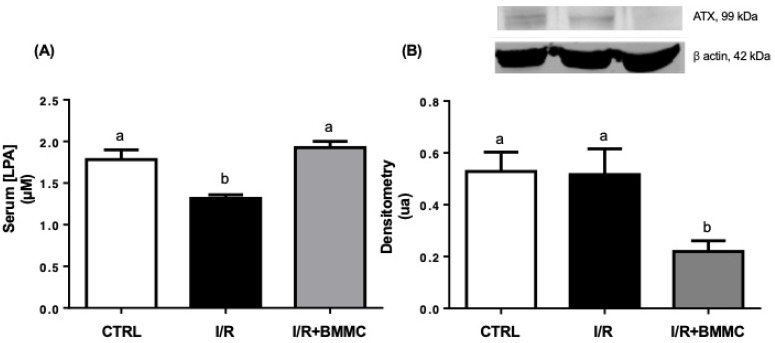
BMMC treatment restored serum LPA levels in I/R rats. (**A**) Serum LPA concentration (μM). (**B**) Autotaxin (ATX) protein expression in kidney tissue. Western blot analysis was performed using SDS-PAGE (12%) with primary antibody anti-ENPP2 (1:500), followed by anti-mouse secondary antibody (1:5000). Upper panel: representative Western blotting and lower panel: densitometric analysis. Data are presented as mean ± SEM (*n* = 10; *p* < 0.05, Tukey’s test). Different letters (“a” and “b”) indicate statistically significant differences.

**Figure 2:**
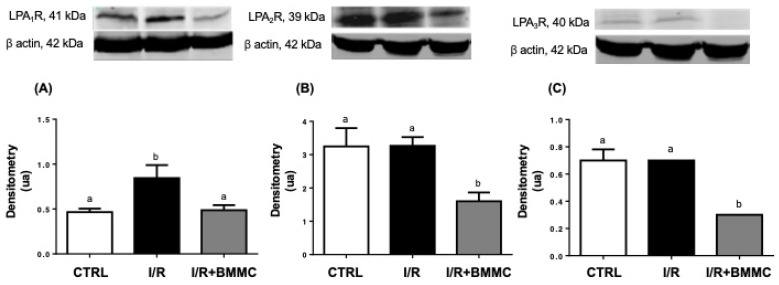
BMMC treatment modulated LPA receptor expression toward a protective profile in I/R rats. (**A**) LPA1R; (**B**) LPA2R and (**C**) LPA3R protein expression in kidney cortex. Western blot analysis was performed using SDS-PAGE (12%) with primary antibodies anti-EDG2 (LPA1R, 1:1000), anti-EDG4 (LPA2R, 1:500), and anti-EDG7 (LPA3R, 1:1000), followed by anti-rabbit secondary antibody (1:5000). Upper panels: representative Western blotting and lower panels: densitometric analysis. Data are expressed as mean ± SEM (*n* = 10; *p* < 0.05, Tukey’s test). Different letters (“a” and “b”) indicate statistically significant differences.

Renal I/R led to a ~30% reduction in plasma LPA levels, while BMMC treatment restored these levels to values comparable to those from the CTRL group (Figure 1A). Concomitantly, BMMC administration downregulated kidney autotaxin (ATX) protein content (Figure 1B). In parallel, I/R caused a 45% increase in LPA_1_ receptor expression in renal tissue, which was prevented by BMMC treatment (Figure 2A). BMMC also reduced the expression of LPA_2_ and LPA_3_ receptors (Figure 2B and Figure 2C, respectively), suggesting an attempt to normalize LPA receptor levels, thus improving and stabilizing LPA signaling.

### 2.2. BMMC Treatment Reduces Accumulation of Nitrogenous Metabolites and Proteinuria Induced by I/R

**Figure 3:**
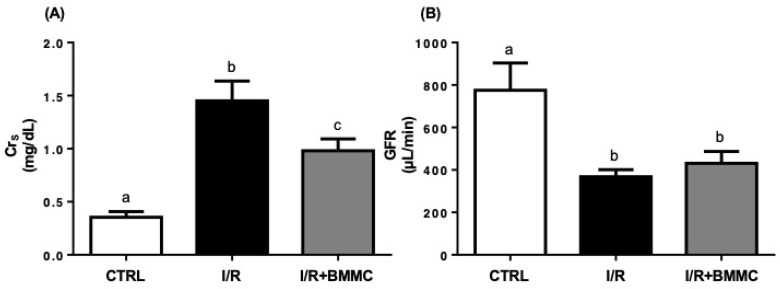
BMMC treatment partially prevented serum creatinine increase but did not improve GFR. (**A**) Serum creatinine (Crs, mg/dL). (**B**) Glomerular filtration rate (GFR, μL/min). Serum creatinine was measured using a commercial kit. GFR was calculated as described in the Section 4. Data are shown as mean ± SEM (*n* = 7; *p* < 0.05, Tukey’s test). Different letters (“a”, “b” and “c”) indicate statistically significant differences. The outliers were excluded according to Hair et al., 2009 [35].

As expected, renal I/R significantly elevated serum creatinine levels (CTRL: 0.35 ± 0.05; I/R: 1.45 ± 0.18 mg/dL; *p* < 0.05), while BMMC treatment partially mitigated this increase (I/R + BMMC: 0.98 ± 0.11 mg/dL; Figure 3A). This protective effect was also evident in the urinary-to-serum creatinine ratio (CrU/CrS), where BMMC limited the I/R-induced decline (CTRL: 211 ± 10.3; I/R: 31 ± 2.1; I/R + BMMC: 58 ± 4.3; *p* < 0.05).

Despite these positive effects, BMMC did not restore glomerular filtration rate (GFR), which remained reduced in both I/R groups (Figure 3B). This was attributed to the persistent decrease in CrU/CrS, which was not compensated by the increased urine volume used in GFR estimation.

**Figure 4:**
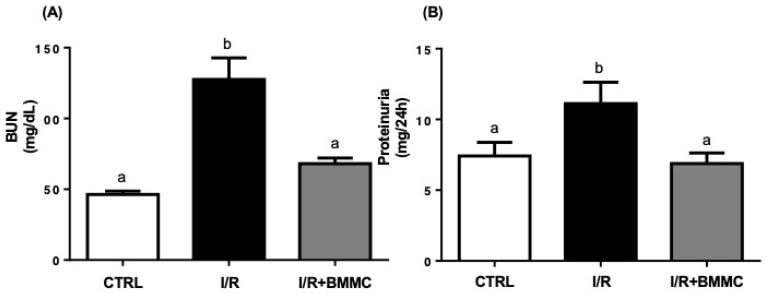
BMMC treatment reduced blood urea nitrogen (BUN) accumulation and proteinuria in I/R rats. (**A**) BUN (mg/dL). (**B**) Proteinuria. Measurements were performed using commercial kits according to the manufacturer’s instructions. Data are presented as mean ± SEM (*n* Ctrl = 7; *n* I/R = 10; *n* I/R + BMMC = 10; *p* < 0.05, Tukey’s test). Different letters (“a” and “b”) indicate statistically significant differences.

Blood urea nitrogen (BUN) levels were significantly elevated after I/R (CTRL: 46.33 ± 2.34; I/R: 127.6 ± 15.24 mg/dL), whereas BMMC treatment effectively maintained BUN values near control levels (Figure 4A). Similarly, I/R-induced proteinuria (11.12 ± 1.51 mg/24 h) was significantly reduced by BMMC treatment (6.88 ± 0.75 mg/24 h; *p* < 0.05), restoring it to levels comparable to the control ones (Figure 4B).

### 2.3. BMMC Partially Restores Electrolyte Handling Following I/R

**Figure 5:**
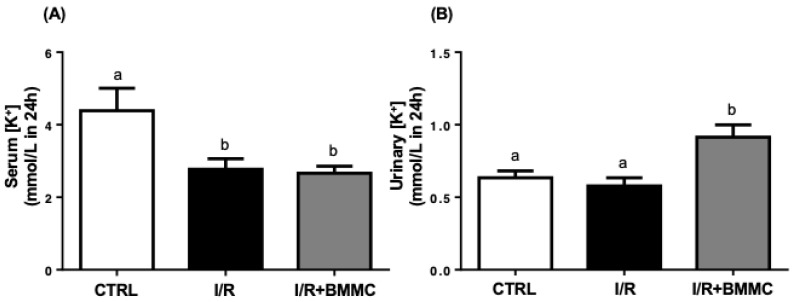
BMMC treatment partially attenuated hypokalemia induced by I/R. (**A**) Serum K^+^ concentration (mmol/L/24 h) and, (**B**) urinary K^+^ excretion (mmol/L/100 g/24 h). Measurements were performed using commercial kits. Data are expressed as mean ± SEM (*n* = 10; *p* < 0.05, Tukey’s test). Different letters (“a” and “b”) indicate statistically significant differences.

Serum potassium (K^+^) levels were markedly reduced after I/R (CTRL: 4.4 ± 0.7; I/R: 2.8 ± 0.3; I/R + BMMC: 2.7 ± 0.2 mmol/L; Figure 5A), indicating hypokalemia in both I/R groups. As expected, BMMC-treated rats showed a significant increase in urinary K^+^ concentration (CTRL: 0.6 ± 0.05; I/R: 0.6 ± 0.06; I/R + BMMC: 0.9 ± 0.08 mmol/L/100 g; Figure 5B).

**Figure 6:**
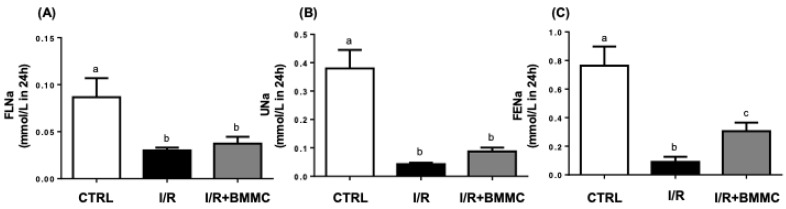
BMMC treatment partially prevented the increase in tubular Na^+^ reabsorption in I/R rats. (**A**) Filtered Na^+^ load (mmol/min). (**B**) Urinary Na^+^ excretion. (**C**) Fractional Na^+^ excretion (%). Serum Na^+^ was measured using a commercial kit, and filtered load and fractional excretion were calculated as described in the Section 4. Data are presented as mean ± SEM (*n* = 9; *p* < 0.05, Tukey’s test). Different letters (“a”, “b” and “c”) indicate statistically significant differences. The outliers were excluded as described [35].

As serum sodium (Na^+^) levels remained unchanged, filtered Na^+^ load (FLNa) was directly proportional to the decreased GFR. I/R reduced FLNa by two-thirds compared to control, and BMMC treatment did not restore this parameter (CTRL: 0.09 ± 0.02; I/R: 0.03 ± 0.003; I/R + BMMC: 0.04 ± 0.007 mmol/L; Figure 6A). Sodium excretion (UNa) was also significantly decreased in I/R rats and remained low in BMMC-treated rats (CTRL: 0.38 ± 0.06; I/R: 0.04 ± 0.005; I/R + BMMC: 0.09 ± 0.01 mmol/L; Figure 6B). The fractional excretion of sodium (FENa) was reduced by 66% in I/R and partially improved by BMMC treatment (CTRL: 0.76 ± 0.13; I/R: 0.09 ± 0.03; I/R + BMMC: 0.31 ± 0.06 mmol/L; *p* < 0.05; Figure 6C).

## 3. Discussion

In this study, we demonstrate that the subcapsular administration of bone marrow-derived mononuclear cells (BMMC) during renal ischemia–reperfusion (I/R) attenuates injury by modulating lysophosphatidic acid (LPA) signaling. Specifically, BMMC restored plasma LPA levels, downregulated kidney autotaxin (ATX), and modulated LPA receptor expression—preventing the I/R-induced upregulation of LPA1R and reducing LPA2R and LPA3R levels. These molecular effects were associated with preserved glomerular function, partial protection against electrolyte imbalance, and a significant reduction in proteinuria, which allow us to hypothesize a protective action of BMMC administration in the maintenance of the correct assembly and composition of the glomerular filtration barrier. As previously reported [36], subcapsular administration of BMMCs after I/R-induced AKI promoted marked structural and functional renal protection, including tubular cell proliferation, reduced apoptosis, decreased inflammatory response, restore mitochondrial function, ATP synthesis with concomitantly preservation of renal histology, plasma creatinine, and urinary osmolality. Altogether, demonstrating the renoprotective function of BMMC intracapsular injection. Here we add new possibilities for BMMC action, mainly the LPA-induced cellular responses.

We used a validated bilateral I/R model that reproduces the key features of early-stage acute kidney injury (AKI), including reduced glomerular filtration rate (GFR), elevated blood urea nitrogen (BUN), creatinine accumulation, and polyuria. This rat model of I/R promoted a mild tubular injury, characterized by both glomerular and tubular damage, mainly in the medulla [29,30,31,32]. The transient polyuria observed is characteristic of the early phase of AKI in rodents. Patients who develop AKI may experience oliguria or anuria (urine volume < 400 mL/24 h) [33]. A possible explanation is that in the experimental methodology applied, the I/R injury was not yet fully established. In the initial phase of the disease, polyuria occurs by decreasing the reabsorption of urea in the collecting duct (in an inefficient attempt to eliminate the accumulation of BUN), making it difficult to preserve the concentrated medullary interstitium and thus, the urine concentration process. The augmented urine volume was due to the kidney’s inability to concentrate the urine [33]. In rats, these changes are easily detected once both kidneys have been clamped. Taken together, the polyuria observed in our model is due to the inability to concentrate urine but not due to hyperfiltration since GFR is decreased. BMMC treatment did not fully protected GFR and normalized urine volume indicating that kidney’s ability to concentrate urine is maintained jeopardized, despite normal BUN.

In the establishment of AKI, the kidneys lose the ability to properly regulate the acid-base equilibrium and hydroelectrolytic balance [34]. Although urinary pH did not significantly differ among groups, slight variations (7.2–7.7) in this logarithmic scale may indicate meaningful shifts in acid-base status. Our findings suggest that I/R leads to urinary acidosis and hypokalemia, while BMMC treatment, paradoxically, intensified urinary K^+^ excretion and caused urinary alkalosis within the first 24 h, possibly reflecting early tubular dysfunction or a transitional homeostatic response.

Many research works, in the literature, aim to demonstrate that cell therapy leads to tissue regeneration [36,37,38,39,40,41,42], but little is known whether this regeneration effectively leads to the total recovery of renal function: the filtration process and the tubular Na^+^ transport, which controls most hydroelectrolyte homeostasis [43]. We evaluated two gold standard metabolites (Crs and BUN) to ascertain whether the BMMC protects glomerular function. Despite the BMMC-induced improvement in glomerular function—evident by lower serum creatinine, reduced BUN, and prevention of proteinuria—GFR remained compromised. This may be explained by the short observation window (24 h) and persistent tubular damage, particularly affecting sodium handling. BMMC failed to restore UNa and FENa, highlighting that tubular transport processes are more resistant to early intervention and may require longer recovery periods.

Importantly, these results are in line with previous studies suggesting that while BMMC therapy reduces fibrosis, cell death, and inflammation [36,37,38,39,40,41,42], its effects on functional tubular recovery remain limited. This dissociation underscores the need for more precise evaluation of both structural and functional recovery in kidney regenerative strategies.

The paracrine action of BMMC remains the most plausible mechanism of action. While VEGF and IGF-1 are well-established paracrine mediators, we also showed that the presence of the BMMC in the damaged kidney promoted a shift in the pattern of some important bioactive lipids, as the levels of sphingosine-1-phosphate [38]. LPA may represent an additional and underexplored factor contributing to kidney repair.

LPA can be an important possible mediator of the BMMC infusion effects due to the following: (i) during the tissue injury, there is an increase in the PLC activity, resulting in an increase in diacylglicerol, which is one possible substrate for the LPA production [44,45]; (ii) LPA and its receptors are directly associated with the progress or interruption of fibrosis [16,17]; (iii) in mesenchymal cells, LPA inhibits the endoplasmic reticulum stress, increasing their viability when transplanted in an environment that is not appropriate to their growth, such as an ischemic tissue [46,47]; and (iv) in the unilateral ureteral obstruction model, despite that LPA levels do not change, the increase in the content of the LPA1R, which is pro-fibrotic, was reported, while treatment with BMMC reverses this effect [38].

In this work, we evaluated the serum LPA concentration in the three experimental groups. During renal I/R the serum LPA concentration decreases approximately 35%. The plasma LPA levels may vary in acute and chronic kidney disease. In chronic kidney disease, augmented plasma LPA levels are related to abnormal renal tubular epithelial cell architecture, recruitment of immune cells to the site of injury, and profibrotic profile [16]. In the mouse model of diabetic renal disease, the LPA1R/LPA3R antagonism attenuated glomerular sclerosis and tubule-interstitial fibrosis development [48,49,50,51,52]. In a mice model of sepsis-inducing AKI, it has been described that LPA plasma levels may be augmented [50] or not altered associated to diminished LPA kidney levels [53]. We showed that I/R-inducing AKI decreases LPA plasma levels, leading to the hypothesis that BMMC through the paracrine bioactive molecules secreted could be a good alternative tool to maintain normal LPA circulating levels. These above related discrepancies may be explained by: (i) the nature of the renal injury, since ischemia/reperfusion is characterized by abrupt tubular and vascular injury, whereas sepsis and chronic disease are driven mainly by systemic inflammation or progressive fibrotic remodeling; (ii) different observation windows, as plasma LPA dynamics may vary between acute and late phases of kidney injury; and (iii) experimental heterogeneity across models, species, and methods of LPA quantification.

The observed modulation of LPA signaling suggests a potential mechanism for BMMC-mediated protection. LPA is a bioactive lipid involved in inflammation, fibrosis, and cell survival, acting via specific G-protein-coupled receptors (LPA1–5). We previously showed that LPA1R expression increases in a chronic renal injury model [38], while others have reported LPA involvement in promoting fibrosis, apoptosis, and epithelial dysfunction in kidney disease [17,19,49,54].

Here we showed that following I/R induced AKI, circulating LPA levels decreased, despite a regular expression of ATX in kidney tissue. Interestingly, subcapsular administration of BMMCs restored circulating LPA levels, even in the presence of reduced ATX protein content in the kidney. Kanehira and colleagues [55] elegantly demonstrated that BMMCs can produce autotaxin (ATX), a key enzyme in LPA synthesis, in response to myeloma cells. These findings led us to hypothesize that ATX derived from BMMCs contributed to the restoration of circulating LPA levels, which in turn may have induced downregulation of kidney ATX expression. This suggests that BMMC may themselves contribute to circulating LPA pools or modulate its metabolism indirectly via secreted bioactive factors, like ATX [10,55]. LPA replenishment mitigates several hallmarks of either I/R- and sepsis-induced AKI [56,57,58]. The I/R provoked kidney LPA1R upregulation which is prevented by the BMMC treatment [38]. In addition to the normal LPA1R, BMMC treatment downregulated LPA2R and LPA3R protein content in the kidney.

Our findings strengthen the hypothesis that modulation of lipid signaling, particularly through LPA and its receptors, is a critical component of the protective effects observed following BMMC administration.

## 4. Material and Methods

### 4.1. Animal Care and Ethics

Adult male Wistar rats were obtained from the Central Animal Facility of the Federal University of Rio de Janeiro (RJ, Brazil). Animals were healthy and immunocompetent, certified by a licensed veterinarian before the experiments. All rats used in this study were non-genetically modified, of standard genotype, and had not undergone any previous experimental procedures. The rats were maintained under controlled temperature (23 ± 2 °C), with a 12 h light/dark cycle, and free access to standard chow (Labina^®^, Purina Agribrands, Paulínia, SP, Brazil) and filtered water. No preregistered protocol was deposited in a public repository. However, all experimental procedures were conducted according to the protocol approved by Institutional Committee for Ethics in Animal Experimentation (protocol IBCCF 147), which included the research question, key design features, and planned analyses in accordance with national guidelines and international standards for the care and use of laboratory animals.

### 4.2. Kidney Ischemia/Reperfusion (I/R) Model

The renal I/R model was based on the protocol described by Benítez-Bribiesca et al. [59], with modifications according to previous work by our group [36,58]. We used the G Power software (version 3.1.9.7) to calculate a priori the n size, considering the significance level as 0.5, the statistic power 0.80. Outliers were recognized according to Hair et al., 2009 [35]. Outliers were defined as values exceeding 2.5 standard deviations from the mean (or 4 SD in some cases), according to the z-score criterion. Thirty male Wistar rats were equally and simple randomly divided into three experimental groups: control (CTRL), I/R and I/R + BMMC. Confounders factors were not controlled. The primary outcome measures were presented in Table 1. The outcome measures assessed kidney function: serum creatinine (a marker of GFR), BUN, proteinuria and electrolytes (Na^+^ and K^+^). One of the authors (LSL) was aware of the group allocation at the different stages of the experiment. For better understanding, the n number (the unit considered as a single rat) is included in each legend of the figures.

Control (CTRL): sham-operated animals underwent surgical manipulation without vascular clamping.

I/R group: rats were anesthetized with xylazine (5 mg/kg, Bayer S.A., São Paulo, Brazil) and ketamine (50 mg/kg, Cristália, Itapira, Brazil) and subjected to bilateral renal artery occlusion with non-traumatic vascular clamps for 30 min, followed by 24 h of reperfusion.

I/R + BMMC group: rats received a subcapsular injection of BMMC (1 × 10^7^ cells in saline) into both kidneys at the onset of reperfusion.

After 24 h, kidneys were excised, placed on ice, and homogenized in an ice colded buffer containing: 250 mM sucrose, 10 mM HEPES-KOH pH 7.4, 2 mM EGTA, 0.15 mg/mL trypsin inhibitor (all from Sigma-Aldrich, St. Louis, MO, USA). Cortical tissue was isolated using iridectomy scissors and sliced using a Stadie-Riggs microtome. Tissue was homogenized in 10 mL of buffer solution with a Teflon-glass homogenizer and centrifuged at 600× *g* for 10 min at 4 °C. The supernatant was collected for further analyses.

To minimize pain, suffering, and distress, all surgical procedures were performed under general anesthesia with ketamine/xylazine (80/10 mg/kg, i.p.), and body temperature was maintained at 37 ± 0.5 °C using a heating pad. The rats were monitored daily for clinical signs of discomfort (piloerection, reduced mobility, abnormal posture, or reduced food and water intake). No unexpected adverse events were observed during the study. Humane endpoints were established in accordance with institutional guidelines, including >20% body weight loss, severe lethargy, persistent self-mutilation, or inability to access food and water. Animals reaching these criteria would be humanely euthanized by overdose of anesthetics (thiopental 200 mg/kg, i.p.). None of the animals met the predefined human endpoints.

### 4.3. BMMC Isolation and Administration

Bone marrow-derived mononuclear cells (BMMCs) were isolated from femurs and tibias of male Wistar rats by flushing with saline, followed by density gradient centrifugation using Ficoll (Science Pro, São Caetano do Sul, Brazil), as previously described [34] and adapted by Beiral et al. [36]. The resulting BMMC suspension was washed twice in fresh medium, and 1 × 10^7^ cells were injected subcapsularly into both kidneys at the onset of reperfusion in the I/R + BMMC group [58,60,61]. The same volume of saline was administered to CTRL and I/R rats.

### 4.4. Western Blotting

Kidney homogenates were incubated in lysis buffer (1% Triton X-100, 50 mM Tris, 30 mM sodium pyrophosphate, 150 mM NaCl, 50 mM NaF, 1 mM sodium orthovanadate) supplemented with protease inhibitors (Roche, Rio de Janeiro, RJ, Brazil). After centrifugation at 300× *g* for 30 min, supernatants were collected and protein concentration was determined using the phenol-pholin method [62]. Proteins were solubilized in Laemmli buffer [63], resolved by SDS-PAGE (12% polyacrylamide gels), and transferred to nitrocellulose membranes (Hybond, Amersham Biosciences, Buckinghamshire, UK). Membranes were blocked in TBS-T (0.05% Tween 20) containing 5% non-fat milk and incubated with primary antibodies as specified in the figure legends.

### 4.5. Plasma LPA Quantification

Plasma LPA levels were quantified using the LPA Assay Kit II (Echelon Biosciences Inc., Salt Lake City, UT, USA) according to the manufacturer’s instructions.

### 4.6. Assessment of Renal Function

Immediately after surgery and during the 24-h reperfusion period, rats were placed in metabolic cages (12 h light/dark cycle, 22–23 °C) with free access to food and water. Urine was collected for 24 h for further analysis. Blood was drawn at the time of euthanasia. Renal function parameters were calculated as previously described by Cortes et al. [61].

### 4.7. Statistical Analysis

Results are presented as mean ± SEM. Data were analyzed by one-way analysis of variance (ANOVA) followed by Tukey’s post hoc test using GraphPad Prism 5.0 software (GraphPad Inc., La Jolla, CA, USA). The assumption of normality was assessed using the Shapiro–Wilk test, and all data met this criterion. Differences were considered statistically significant at *p* < 0.05, as indicated by the different letters in the figures, and explained in the respective captions.

## 5. Conclusions

We show that renal I/R injury significantly alters kidney structure and function and leads to abnormal LPA signaling. The subcapsular administration of BMMC during I/R exerts beneficial effects on renal outcomes, likely through the paracrine modulation of LPA signaling pathways. BMMC treatment restored plasma LPA levels, reduced ATX expression, and normalized LPA receptor patterns. These effects were associated with improved glomerular function and partial preservation of electrolyte and protein handling. However, tubular recovery remained incomplete, emphasizing the complexity of renal repair and the need for further research. Future studies should explore long-term outcomes, the contribution of other paracrine mediators, and the potential of combining BMMC therapy with pharmacological modulation of the LPA axis.

## Figures and Tables

**Table 1:** General renal physiological parameters.

	CONTROL *n* = 10	I/R *n* = 10	I/R + BMMC *n* = 10
Weight (g)	185.4 ± 13.47 ^a^	171.1 ± 9.23 ^a^	165.0 ± 4.35 ^a^
Water intake (mL/100 g)	6.5 ± 1.62 ^a^	6.2 ± 0.72 ^a^	8.9 ± 0.97 ^a^
Urinary pH	7.4 ± 0.31 ^a^	7.2 ± 0.38 ^a^	7.6 ± 0.27 ^a^
Urinary vol. (mL/100 g/24 h)	3.7 ± 0.34 ^a^	7.7 ± 1.03 ^b^	7.4 ± 1.06 ^b^

Rats were subjected to bilateral ischemia for 30 min followed by 24 h reperfusion (I/R) or to sham surgery. During ischemia, I/R + BMMC rats received a single dose (107 cells in saline) administered intracapsularly. Urine collections were taken at the end of the 24 h reperfusion, corresponding to those in which renal function parameters were measured. Values are means ± SEM. Different superscripted lower-case letters “a” and “b” indicate statistically significant differences (*p* < 0.05; one-way ANOVA followed by Tukey’s post-test).

## Data Availability

Raw data are available with the authors, if considered necessary.
